# The histamine H3R antagonist DL77 attenuates autistic behaviors in a prenatal valproic acid-induced mouse model of autism

**DOI:** 10.1038/s41598-018-31385-7

**Published:** 2018-08-30

**Authors:** Nermin Eissa, Petrilla Jayaprakash, Sheikh Azimullah, Shreesh K. Ojha, Mohammed Al-Houqani, Fakhreya Y. Jalal, Dorota Łażewska, Katarzyna Kieć-Kononowicz, Bassem Sadek

**Affiliations:** 1 Department of Pharmacology & Therapeutics, College of Medicine & Health Sciences, United Arab Emirates University, P.O. Box 17666, Al Ain, UAE; 2 Department of Internal Medicine, College of Medicine and Health Sciences, United Arab Emirates University, P.O. Box 17666, Al Ain, UAE; 3 Jagiellonian University-Medical College, Faculty of Pharmacy, Department of Technology and Biotechnology of Drugs, Medyczna 9 St., 30-688 Kraków, Poland

## Abstract

Autistic spectrum disorder (ASD) is a neurodevelopmental disorder characterized by impairment in social communication and restricted/repetitive behavior patterns or interests. Antagonists targeting histamine H3 receptor (H3R) are considered potential therapeutic agents for the therapeutic management of different brain disorders, e.g., cognitive impairments. Therefore, the effects of subchronic treatment with the potent and selective H3R antagonist DL77 (5, 10, or 15 mg/kg, i.p.) on sociability, social novelty, anxiety, and aggressive/repetitive behavior in male Tuck-Ordinary (TO) mice with ASD-like behaviors induced by prenatal exposure to valproic acid (VPA, 500 mg/kg, i.p.) were evaluated using the three-chamber test (TCT), marble burying test (MBT), nestlet shredding test (NST), and elevated plus maze (EPM) test. The results showed that VPA-exposed mice exhibited significantly lower sociability and social novelty preference compared to VPA-exposed mice that were pretreated with DL77 (10 or 15 mg/kg, i.p.). VPA-exposed mice presented a significantly higher percentage of buried marbles in MBT and shredded nestlet significantly more in NST compared to the control groups. However, VPA-exposed animals pretreated with DL77 (10 or 15 mg/kg, i.p.) buried a reduced percentage of marbles in MBT and presented a significantly lower percentage of shredding behavior in NST. On the other hand, pretreatment with DL77 (5, 10, or 15 mg/kg, i.p.) failed to restore the disturbed anxiety levels and hyperactivity observed in VPA-exposed animals in EPM, whereas the reference drug donepezil (DOZ, 1 mg/kg, i.p.) significantly palliated the anxiety and reduced the hyperactivity measures of VPA-exposed mice. Furthermore, pretreatment with DL77 (10 or 15 mg/kg, i.p.) modulated oxidative stress status by increasing GSH and decreasing MDA, and it attenuated the proinflammatory cytokines IL-1β, IL-6 and TNF-α exacerbated by lipopolysaccharide (LPS) challenge, in VPA-exposed mouse brain tissue. Taken together, these results provide evidence that modulation of brain histaminergic neurotransmission, such as by subchronic administration of the H3R antagonist DL77, may serve as an effective pharmacological therapeutic target to rescue ASD-like behaviors in VPA-exposed animals, although further investigations are necessary to corroborate and expand these initial data.

## Introduction

Autistic spectrum disorder (ASD) is a neurodevelopmental brain disorder characterized by two major core behavioral symptoms: impairment in social interaction and communication and restricted/repetitive behavior patterns or interests, observed often before the age of three years and lasting throughout life^1,2^. Evidence suggests that genetic and environmental factors increase the incidence of ASD in children^1^. ASD has become a high priority for scientists and has attracted the public’s attention because of the reported increase in its prevalence, which varies across studies but it is estimated to be 1 in 160 children worldwide and appears to have occurred globally^3^. The heterogeneity of clinical and behavioral symptoms in autistic children is part of the difficulty in understanding the pathophysiology of this disorder. Consequently, no specific treatment can be effective for all autistic children. Notably, cognitive problems are the most frequently reported features by parents of children with ASD, with prevalence estimates of 44–83% for these features in this population, underscoring the importance of recognizing these conditions in children with ASD as well as the need for more systematic research as an initial step in developing treatment strategies^4^. Importantly, behaviors inherent to ASD, such as impairments in communication, stereotype, aggression and attention-deficit hyperactivity disorder (ADHAD), may be exacerbated by increased memory disturbances^1,2,5^. Therefore, a successful pharmacological intervention strategy will not only palliate the impact of these symptomatic behaviors but also treat conditions that affect the ability of children to compensate for their deficits. In the last two decades, there has been a growing interest in the study of histamine in the CNS and its influence on behavior in both physiological conditions and brain disorders^5^. The histaminergic system is involved in basic physiological functions, such as the sleep-wake cycle, energy and endocrine homeostasis, sensory and motor functions, cognition, and attention, which are all severely affected in neuropsychiatric disorders^6–9^. Thus, the brain histaminergic system is an attractive pharmacological target for therapeutic purposes, and many efforts have been made to develop drugs that could act on different histamine receptors (H1R, H2R, H3R and H4R)^9,10^. However, there are few studies related to the use of histamine receptor antagonists to treat autistic behavior. In 1997, it was proposed that famotidine, an antagonist of histamine H2R, would be a potential treatment for children with ASD^11^. Famotidine has improved sociability in a patient diagnosed with schizophrenia (SCH)^12^, a disorder that shares various symptoms and genetic factors with ASD^13,14^. Later, famotidine was tested in a group of children with ASD, and 44% of them presented evidence of behavioral improvement. Moreover, the histamine H1R antagonist niaprazine has ameliorated symptoms such as unstable attention, resistance to change and frustration in patients with ASD^15^. Furthermore, it has been suggested that ligands of histamine H3R are considered potential therapeutic agents for the treatment of Alzheimer’s disease (AD), SCH, and narcolepsy^16–18^. In an animal model of SCH, the use of an H3R antagonist ameliorates behavioral impairments^19^, including spatial working memory deficit, an abnormality also found in patients with ASD^6^. Antagonism of H3R can attenuate impaired social behavior in rodents exposed to phencyclidine, a finding that may also have implications for ASD^9^. Interestingly, the old-generation H3R antagonist ciproxifan attenuates at least some sociability deficits and stereotypies present in the animal model of autism induced by valproic acid (VPA)^1^. Notably, embryonic exposure to VPA causes several molecular and neurochemical changes in zebrafish, which continue into adulthood and accompany impaired sociability, demonstrating the possible association between brain histaminergic system and the outcomes related to neuropsychiatric disorders^20^. In the postmortem dorsolateral prefrontal cortex of 13 human subjects with ASD, significant alterations in the expression of key histamine genes, including all HR subtypes H1-4Rs, have been observed, signifying the involvement of modulated histaminergic signaling in the brain of ASD subjects^21^. The H3Rs are important modulators of numerous central control mechanisms^22^. Therefore, blockade of inhibitory histamine H3 auto-receptors reinforces histaminergic neurotransmission, while antagonism of H3 hetero-receptors accelerates the corticolimbic liberation of acetylcholine, norepinephrine, glutamate, dopamine, serotonin and gamma-aminobutyric acid (GABA)^7,9,12^. Considering these findings, we evaluated the effects of the novel potent and selective H3R antagonist DL77, which has confirmed high *in vitro* antagonist affinity in the subnanomolar range (p*K*_i_ = 8.08)^23–25^, high *in-vivo* H3R antagonist central potency (ED_50_ = 2.1 ± 0.2 mg/kg, per os)^23–25^, and an excellent selectivity profile (p*K*_i_ (hH4R) = 4.31; p*K*_i_ (hH1R) = 6.18; p*K*_i_ (α2R) = 2.10)^23–26^ on the ASD-like behavioral deficits in mice. The non-imidazole H3R antagonist DL77 was tested in the current experiments because it has shown promising anticonvulsant and procognitive effects in different memory models in rodents^24,27^. DL77 (3–30 mg/kg, i.p.) has also exhibited encouraging effects on alcohol intake and preference in adult C57BL/6 mice^25^. Comprehensive as well as objective sociability and social novelty preferences of autistic animals were assessed using the three-chamber test (TCT) paradigm to evaluate the behavioral outcomes triggered by subchronic systemic pretreatment with DL77, applying donepezil (DOZ) as a reference drug. Additionally, the effects of DL77 on repetitive and anxious behavior using the marble burying test (MBT), nestlet shredding test (NST), and elevated plus maze (EPM) test were investigated, since stereotyped repetitive as well as anxiety problems are considered possible predictors of intensified symptoms of ASD. Moreover, the effects of subchronic administration of the H3R antagonist DL77 on the expression of several oxidative stress markers and proinflammatory cytokines were assessed in the cerebellum tissues (histamine H1-4Rs are present in high density)^28^, since neuroinflammation and oxidative stress are also considered possible predictors of intensified ASD-like symptoms in mice prenatally exposed VPA^29^. Furthermore, the abrogative effects of the CNS-penetrant H3R agonist (*R*)-α-methylhistamine (RAMH), the centrally acting H1R antagonist pyrilamine (PYR), and the H2R antagonist zolantidine (ZOL) on the DL77-provided behavioral and biochemical enhancements were assessed to further explain whether brain histaminergic neurotransmission is involved in the effects provided by the H3R antagonist DL77.

## Materials and Methods

### Animals

**Figure 1:**
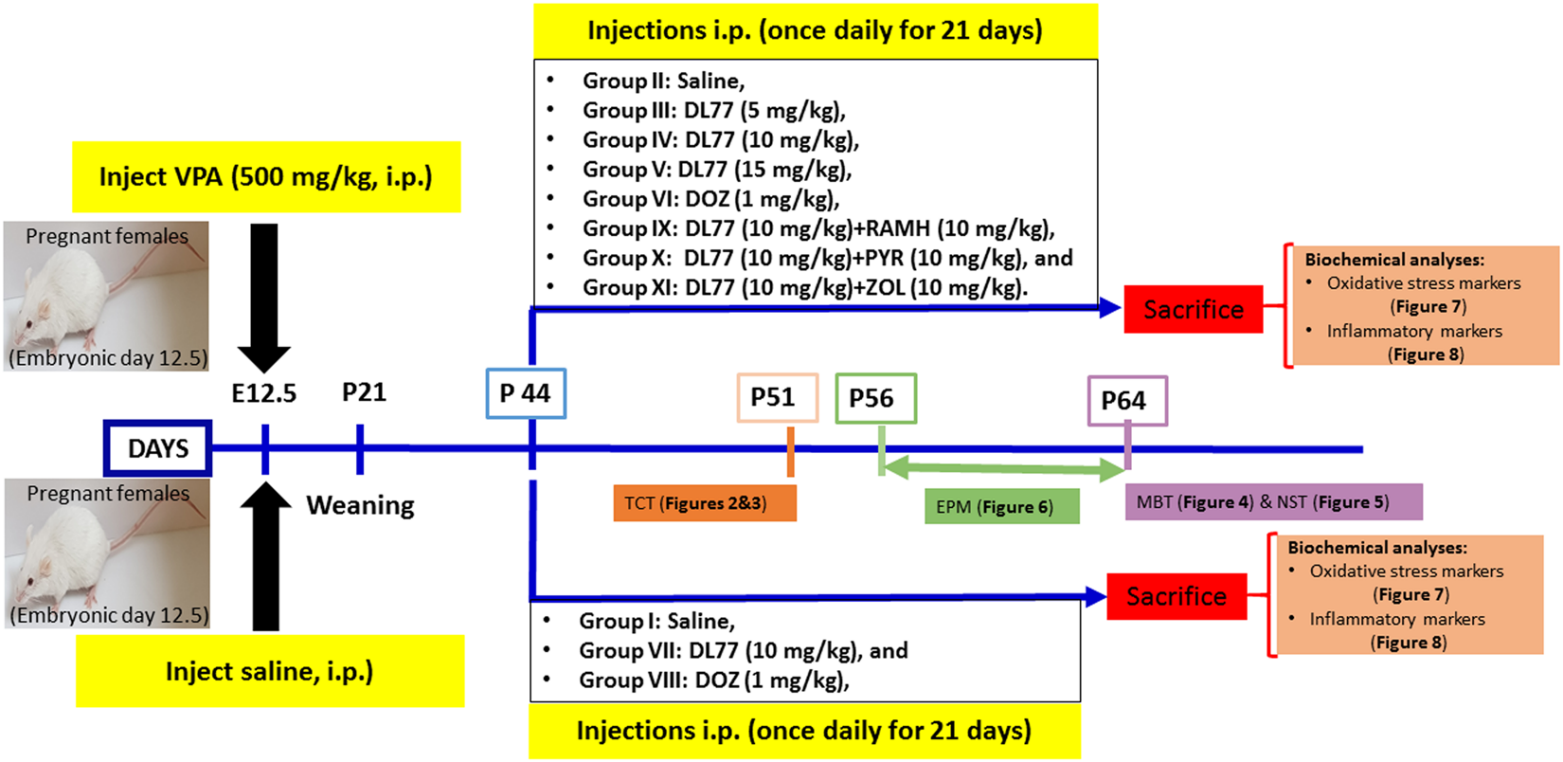
Schematic diagram of drug treatments, behavioral studies, and biochemical assessments with VPA mice. Pregnant mice were injected with VPA (500 mg/kg, i.p.) at embryonic day 12.5 (E12.5). Treatments started from postnatal day (P44). Injections (once daily) continued for 21 days until P64. Behavioral studies were carried starting from P51. All mice were then sacrificed at P64 for biochemical analyses.

Female Tuck-Ordinary (TO) mice (aged 8 weeks, weighing 30–35 g) (Harlan, UK) bred in the local central animal facility of the College of Medicine and Health Sciences, United Arab Emirates University^30^, were mated. Pregnancy was confirmed by the presence of a vaginal plug on embryonic day 0 (E0). They were housed in plastic cages under a standard light/dark cycle (12 h light cycle, lights on 6 a.m.) at constant temperature 22 + 1 °C, with free access to tap water and a standard rodent chow diet. On E12.5, 17 pregnant females were intraperitoneally (i.p.) injected with 500 mg/kg VPA (Sigma-Aldrich, St. Louis, MO, USA) dissolved in isotonic 0.9% sodium chloride solution, and another 5 pregnant females received an equal volume of saline, as previously described^31,32^. Two females died after injection of VPA, and only 12 delivered pups. Pups from VPA-exposed mothers (Group 1) and from mothers that received saline (Group 2) were used in the study. All offspring were weaned, gender-grouped at 3 weeks of age and randomly divided into several cages (5–6 mice/cage). Male offspring only from VPA-exposed mothers (Group 1) and from mothers that received saline (Group 2) were further divided into 2 subgroups, and then the mice in each subgroup were intraperitoneally (i.p.) treated as shown in the schematic experimental design (Fig. 1). The experiments of the current study were carried out between 9:00 am and 3:00 pm, and all procedures were approved by the Institutional Animal Ethics Committee of College of Medicine and Health Sciences/United Arab Emirates University (Approval No. ERA-2017-5603). All authors confirm that all methods were carried out in accordance with relevant guidelines and regulations. To reduce the number of animals used, the levels of oxidative stress and proinflammatory cytokines were studied in the same groups of animals that were subjected to behavioral tests.

### Drugs

The H3R antagonist DL77 [1-(3-(4-*tert*-pentylphenoxy) propyl)piperidine] was designed and synthesized in the Department of Technology and Biotechnology of Drugs Krakow, Poland, according to a previously described procedure^23^. Sodium valproate (VPA) (500 mg/kg, i.p.), donepezil hydrochloride (DOZ) (1 mg/kg, i.p.) (reference drug), the H3R agonist RAMH (10 mg/kg, i.p.), the brain-penetrant H1R antagonist PYR (10 mg/kg, i.p.), the brain-penetrant H2R antagonist ZOL (10 mg/kg, i.p.), LPS (from *E*. *coli* serotype 0111:B4), and the assay kit for GSH were all purchased from Sigma-Aldrich (St. Louis, MO, USA). The lipid peroxidation assay kit for estimation of malondialdehyde (MDA) was obtained from North West Life Science (Vancouver, WA, USA). For estimation of the proinflammatory cytokines IL-1β, IL-6 and TNF-α mediated by lipopolysaccharide (LPS), commercially available ELISA kits were purchased from R&D Systems (Minneapolis, MN, USA). All the reagents used in the study were of analytical grade. All drugs were dissolved in isotonic saline solution and injected intraperitoneally (i.p.) at a volume of 10 ml/kg adjusted to body weight, and all doses are expressed in terms of the free base.

### Treatments

Different doses of DL77 (5, 10, and 15 mg/kg), DOZ 1 mg/kg or vehicle (saline) were injected once daily for 21 days. All doses were selected based on the results of previous studies and are expressed in terms of the free bases^25,33,34^. The H3R antagonists DL77 and DOZ or saline was administered 30–45 minutes before each behavioral test, and a group of 5–7 animals was used for each behavioral test. Behavioral testing was performed between 9:00 am and 3:00 pm in an order randomized by group and in the following sequence once the animals were 50 days old: three-chamber test (TCT), elevated plus maze (EPM) test, marble burying test (MBT) and nestlet shredding test (NST). The subchronic treatment started one week before the behavioral test and was continued for a total of 21 days as described above. Then, the animals were deeply anesthetized with pentobarbital (40 mg/kg, i.p., body weight), and cardiac perfusion was carried out using 0.01 M phosphate-buffered saline (PBS) at pH 7.4 to wash out the blood. The brains were quickly removed and placed on an ice plate, where the two hemispheres were separated. The cerebellum was excised from the brain and snap-frozen in liquid nitrogen for biochemical tests^35^. The animals were injected with LPS (25 µg/kg, i.p.) two hours before the sacrifice according to previously published protocols^29^. All observers who performed the behavioral and biochemical tests were blinded to the experimental groups.

### Study Design

**Figure 2:**
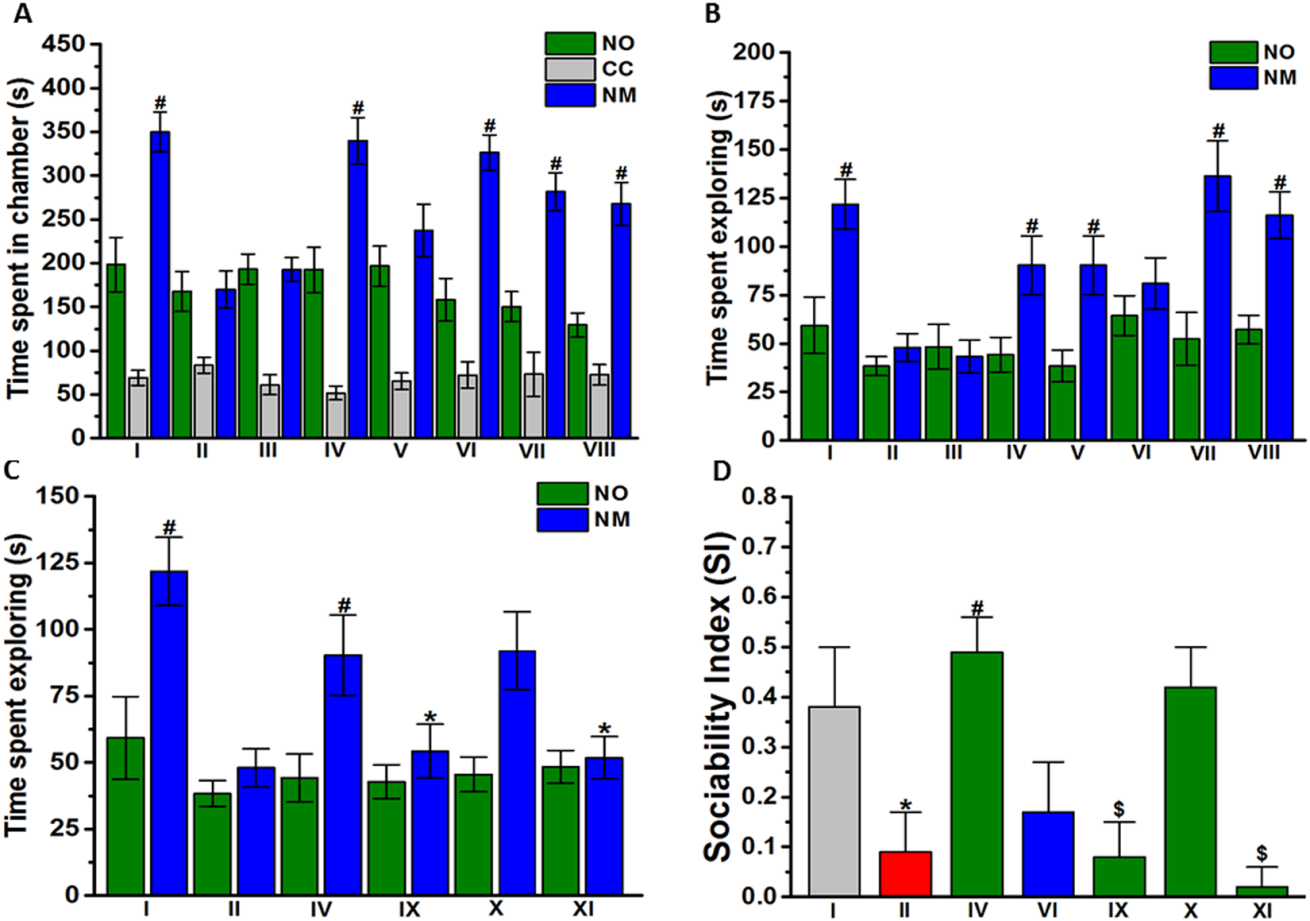
Effects of DL77 and DOZ on sociability assessed in TCT paradigm. After 10 minutes of acclimatization, male subjects were allowed to explore all chambers for 10 min. The results obtained were (**A**) time spent in the chamber of the novel object (NO), the novel mouse (NM), or the central chamber (CC); (**B**) time spent exploring the novel object (NO) or novel mouse (NM); (**C**) abrogative effects of subchronic (21 days) systemic co-administration of RAMH (10 mg/kg, i.p. for IX), PYR (10 mg/kg, i.p. for X), or ZOL (10 mg/kg, i.p., for XI) on the DL77 (10 mg)-provided enhancement of sociability. Saline-exposed mice were injected with saline in group I, DL77 (10 mg/kg, i.p.) in group VII, or DOZ (1 mg/kg, i.p.) in group VIII (**A**,**B**). DL77 (at a dose of 5, 10, or 15 mg/kg in group III, IV, or V, respectively) or DOZ (1 mg/kg, i.p. in groups VI and VIII) was administered subchronically for 21 days. Figure shows mean ± SEM (n = 6–7). ^#^*P* < 0.05 *vs*. time spent in the chamber of NO (**A**–**C**) or *vs*. SI of VPA-exposed mice (group II) (**D**). (**D**) Sociability index (SI). *P < 0.05 *vs*. time spent exploring NM of DL77(10 mg)-treated VPA-exposed mice (group IV) (**C**) or *vs*. SI of (saline)-treated saline-exposed mice (group I) (**D**). ^$^*P* < 0.05 *vs*. DL77(10 mg)-treated VPA-exposed mice (group IV) (**D**).

**Figure 3:**
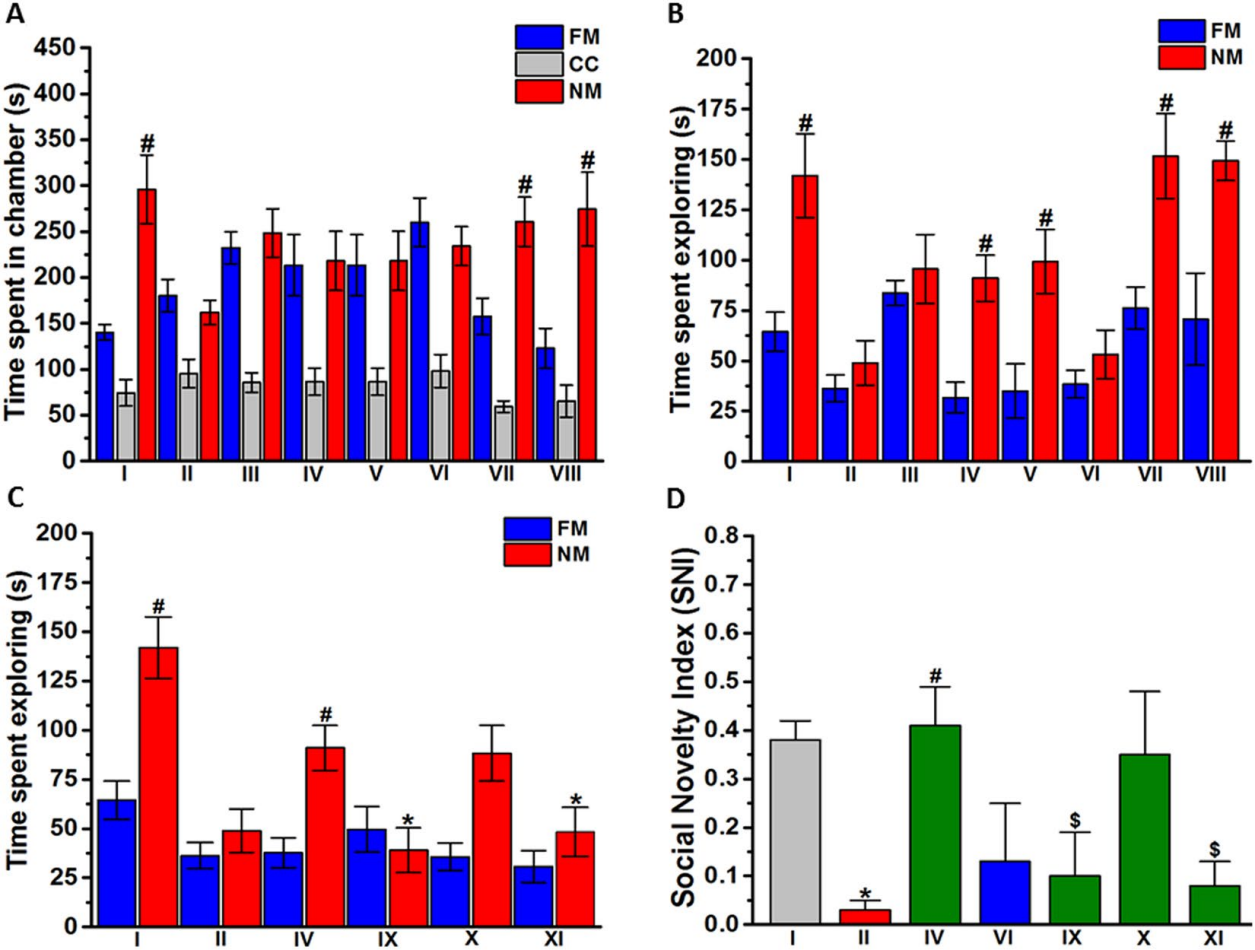
Effects of DL77 and DOZ on social novelty assessed in TCT paradigm. After 10 minutes of acclimatization, male subjects were allowed to explore all chambers for 10 min. The results obtained were (**A**) time spent in the chamber of the familiar mouse (FM), novel mouse (NM), or central chamber (CC); (**B**) time spent exploring the familiar mouse (FM) or novel mouse (NM); (**C**) effects of subchronic (21 days) systemic co-administration of RAMH (10 mg/kg, i.p. for IX), PYR (10 mg/kg, i.p. for X), or ZOL (10 mg/kg, i.p., for XI) on the DL77 (10 mg)-provided enhancement of social novelty. Saline-exposed mice were injected with saline in group I, DL77 (10 mg/kg, i.p.) in group VII, or DOZ (1 mg/kg, i.p.) in group VIII (**A**,**B**). DL77 (at a dose of 5, 10, or 15 mg/kg in group III, IV, or V, respectively) or DOZ (1 mg/kg, i.p. in groups VI and VIII) was administered subchronically for 21 days. Figure shows mean ± SEM (n = 5–7). ^#^*P* < 0.05 *vs*. time spent in the chamber FM (**A**–**C**) or *vs*. SNI of VPA-exposed mice (group II) (**D**). (**D**) Social novelty index (SNI). *P < 0.05 *vs*. time spent exploring NM of DL77(10 mg)-treated VPA-exposed mice (group IV) (**C**) or *vs*. SNI of (saline)-treated saline-exposed mice (group I) (**D**). ^$^*P* < 0.05 *vs*. DL77(10 mg)-treated VPA-exposed mice (group IV) (**D**).

Pregnant TO mice were injected i.p. with either VPA (500 mg/kg, i.p.) (Group 1) or saline (Group 2) on embryonic day 12.5 (E12.5)^36^ and returned to their home cages. VPA was dissolved in isotonic 0.9% NaCl solution (saline), and the volume of injection was 10 ml/kg. Five pregnant mice were exposed to saline and gave birth to an average of 20–30 male offspring. Another group of pregnant mice (n = 17) were exposed to VPA; of that group, 12 gave birth to an average of 48–56 male offspring. All offspring were weaned, sexed at three weeks of age, and then randomly divided into several cages (5–6 mice/cage). Each cage contained mice of both experimental groups (Group 1: prenatally VPA-exposed offspring; Group 2: prenatally saline-exposed offspring). Group 1 was subdivided into nine subgroups of 5–7 VPA-exposed mice as follows: group II: VPA-exposed mice injected with saline; group III: VPA-exposed mice injected with DL77 (5 mg/kg, i.p.); group IV: VPA-exposed mice injected with DL77 (10 mg/kg, i.p.); group V: VPA-exposed mice injected with DL77 (15 mg/kg, i.p.); group VI: VPA-exposed mice injected with DOZ (1 mg/kg, i.p.); group IX: DL77 (10 mg/kg, i.p.) was co-administered with RAMH (10 mg/kg, i.p.); group X: DL77 (10 mg/kg, i.p.) was co-administered with PYR (10 mg/kg, i.p.); group XI: DL77 (10 mg/kg, i.p.) was co-administered with ZOL (10 mg/kg, i.p.) (Figs 2A–D and 3A–D). Group 2 was subdivided into three subgroups of 5–7 saline-exposed mice as follows: group I: saline-exposed mice injected with saline; group VII: saline-exposed mice injected with DL77 (10 mg/kg, i.p.), and group VIII: saline-exposed mice injected with DOZ (1 mg/kg, i.p.) (Figs 2A–D and 3A–D).

### Behavioral tests

#### Three-chamber test (TCT)

TCT assesses cognition in the form of general sociability and interest in social novelty in rodents, identifying rodents with deficits in sociability and/or social novelty. The three-chamber test of social interaction and social novelty recognition was performed as described previously^37^, but with a slight modification in the apparatus, with one center chamber (40 × 20 × 22 cm) and two side chambers (40 × 20 × 22 cm) separated by two sliding doors under a light intensity of ~120 lux. The test was composed of four 10-min sessions. In the first session, a test mouse was habituated to the center chamber with the two doors closed for 5 min. In the second session, the doors were opened and a test mouse was allowed to explore all three chambers for 5 min. Before starting the third session, a stranger mouse (same age and gender with no previous contact with the test mouse), referred to as a novel mouse (NM), was placed in a small plastic cage in the either left or right chamber, chosen randomly to avoid side preference, while the other cage was kept empty in the opposite chamber and was referred to as a novel object (NO). In the third session, the test mouse was allowed to explore all three chambers and cages for 10 min. The time spent in the chamber with NM and around the cage was compared with the time spent in the chamber with NO. Additionally, the time spent exploring the NM and NO (direct contact) was recorded. As previously described, the sociability index (SI) and social novelty preference index (SNI) were evaluated to allow the direct comparison of social behavior of the treated groups^38^. The score for SI and for SNI ranged from 0.02–0.5; as the score became more positive and closer to 1, the more social was the tested animal. The SI was calculated using the following equation: (Fig. 2D)

$$\begin{array}{rcl}{\rm{SI}} & = & [{\rm{Time}}\,{\rm{exploring}}\,{\rm{novel}}\,{\rm{mouse}}\,1-{\rm{Time}}\,{\rm{exploring}}\,{\rm{novel}}\,{\rm{object}}]\\  &  & \div\,[{\rm{Time}}\,{\rm{exploring}}\,{\rm{novel}}\,{\rm{mouse}}\,1+{\rm{Time}}\,{\rm{exploring}}\,{\rm{novel}}\,{\rm{object}}].\end{array}$$

A new stranger mouse was added to the empty cage 10 min after the end of the third session, after which the test mouse was allowed to explore the environment for 10 min, which made up the fourth session. The new stranger mouse was referred to as NM, while the stranger mouse from the previous session was referred to as the familiar mouse (FM). The same parameters, namely, time spent in the chamber and exploratory time, were measured as in the previous session to evaluate the preference of the test mouse to NM over FM in the cage. Similarly, SNI was calculated, and it ranged from 0.02–0.5, where a value closer to 1 indicated that an animal was more interested in social novelty. The SNI was calculated as follows: (Fig. 3D)

$$\begin{array}{rcl}{\rm{SNI}} & = & [{\rm{Time}}\,{\rm{exploring}}\,{\rm{novel}}\,{\rm{mouse}}-{\rm{Time}}\,{\rm{exploring}}\,{\rm{familiar}}\,{\rm{mouse}}]\\  &  & \div\,[{\rm{Time}}\,{\rm{exploring}}\,{\rm{novel}}\,{\rm{mouse}}+{\rm{Time}}\,{\rm{exploring}}\,{\rm{familiar}}\,{\rm{mouse}}].\end{array}$$

The reason for the first session is that it might increase mouse exploration of side chambers relative to the center chamber in sessions 3 and 4. Another objective of habituation in the center chamber before the whole-apparatus habituation was to make the center chamber a familiar “home base” of the test mouse^39^, as reported in the original papers^36,40^. In addition, the time of habituations could be flexible (5–30 min)^39^. Stranger mice (8–16 weeks old) were habituated to the plastic cage in the three-chamber apparatus for 30 minutes 24 h before the test, as described previously^36^. Time spent in the chamber and sniffing were measured using EthoVision^®^ Software (Noldus, Netherlands).

#### Marble burying test (MBT)

MBT behavior is an accurate reflection of repetitive digging behavior^41^. The test was performed as previously reported with slight modifications^41–43^. Briefly, cages (26 cm × 48 cm × 20 cm) were filled with fresh, unscented mouse bedding material to a depth of 5 cm, and the bedding surface was leveled by placing another cage of the same size onto the surface of the bedding. The mouse was individually added for habituation. After 10 min, this mouse was removed, and 20 glass marbles (15 mm diameter) were carefully overlaid equidistantly in a 4 × 5 arrangement. Each mouse was returned to its designated test cage and allowed to explore for 30 min. The number of marbles buried (>50% marble covered by the bedding) was recorded^44,45^.

#### Nestlet Shredding test (NST)

NST is a powerful behavioral assay to assess repetitive and compulsive-like behavior. It was performed by placing one mouse into a cage (19 cm × 29 cm × 13 cm). Fresh, unscented mouse bedding material was added to each cage to a depth of 0.5 cm, and the bedding was packed by placing another cage of the same size onto the surface of the bedding. Commercially available cotton fiber (nestlets) (5 cm × 5 cm, 5 mm thick, ~2.5 g each) was weighed on an analytical balance. One nestlet was placed on top of the bedding in each test cage, and the filter-top cover was placed on the cage. No food and water were given during the test. The mouse was left undisturbed in the cage with the nestlet for 30 minutes. Then, the mouse was then removed and returned to its home cage after test completion. The remaining intact nestlet material was removed from the cage with forceps and allow to dry overnight. The remaining un-shredded nestlet was weighed, and the weight difference was divided by the starting weight to calculate percentage of nestlet shredded. We discarded the remaining shredded nestlet material and bedding^44^.

#### Elevated Plus Maze (EPM) test

The EPM test was performed as previously described^46–49^. Briefly, a four-armed apparatus was elevated 40 cm above the ground. It consisted of two opposite open arms (40 cm × 6 cm) and two opposite closed arms of the same size with high, black-painted walls. The arms were connected by a central square (6 cm × 6 cm). An animal was placed in the center of the maze facing an open arm. The maze was lighted with a 60 W bulb approximately one meter above the maze. The amounts of time spent with the head and forepaws on the open arms and closed arms of the maze as well as the number of entries into each arm were measured over 5 minutes using EthoVision^®^ Software (Noldus, Netherlands). The maze was thoroughly cleaned with a tissue dampened with 70% (volume/volume; v/v) alcohol to remove the odor after each mouse was tested. The total number of entries into the closed arms is usually used as an index of locomotor activity in the test^46–49^.

### Biochemical assessments

#### Enzyme-linked Immunosorbent Assay (ELISA)

ELISA was used to quantify the proinflammatory cytokines, mainly interleukin-1β (IL-1β), interleukin-6 (IL-6), and tumor necrosis factor-alpha (TNF-α), in the cerebellum. Commercially available ELISA kits (DuoSet) for IL-1β, IL-6, and TNF-α were purchased from R&D Systems. The levels of IL-1β, IL-6, and TNF-α were estimated as recommended by the manufacturer’s instructions. After brain extraction, the required tissues (mentioned above) were dissected, quickly frozen separately, and stored at −70 °C. On the day of assay, the neural tissues were homogenized on ice in the extraction buffer recommended by the manufacturer, RIPA buffer (50 mM Tris HCl, pH 7.4, 140 mM NaCl, 1 mM EDTA, 0.5% Triton X-100, 0.5% sodium deoxycholate) with protease and phosphatase inhibitors (product No. 1861281, Thermo Scientific, Waltham, Massachusetts, United States). The homogenates were sonicated and centrifuged for 30 min at 12,500 rpm at 4 °C to remove tissue debris, and the resulting supernatant was used for the proinflammatory cytokine assessment and oxidative stress analysis. For proinflammatory cytokine analysis, the protein concentrations were determined using the Pierce® BCA Protein Assay Reagent Kit (Thermo Fisher Scientific Inc., Rockford, IL, USA). Briefly, 96-well plates were coated with the diluted capture antibody (100 μL) overnight at room temperature. Each well was aspirated and washed with wash buffer (0.05% Tween 20 in PBS 0.01 M pH 7.4). The plate was blocked by adding reagent diluent (1% bovine serum albumin in PBS [300 μL]) for 1 hour and washed with wash buffer. Samples or standards (100 μL) were added to the well and incubated for 2 hours, then washed with wash buffer. Each well received detection antibody (100 μL), was incubated for 2 hours at room temperature, and was washed with wash buffer. The well then received working solution (1:200) of streptavidin horseradish peroxidase (100 μL) and was further incubated for 30–40 minutes and then washed with wash buffer. The wells received substrate solution (100 μL) and incubated for 20 minutes. Finally, stop solution (2N H_2_SO_4_ [50 μL]) was added and mixed by gentle plate tapping. Immediately, the optical density of each well was read at 450 nm using a microplate absorbance reader (Sunrise, TECAN). The results are expressed as pg/mg protein^35,50^.

#### Oxidative stress marker estimations

Lipid peroxidation product: malondialdehyde (MDA): An MDA detection kit (North West Life Science, Vancouver, WA, USA) was used to determine the amount of lipid peroxidation following the manufacturer’s instructions. Briefly, samples or calibrators (250 μL) were incubated in the presence of acid reagent and thiobarbituric acid (250 μL). Each sample was vortexed vigorously. Then, the samples were incubated for 60 minutes at 60 °C and centrifuged at 10,000 × *g* for 2–3 minutes. The reaction mixture was transferred to a cuvette and its spectra at 532 nm recorded. The results are expressed as μM MDA/mg protein.

Glutathione (GSH): For the estimation of GSH, a commercially available GSH kit was used following the manufacturer’s instructions. Briefly, the samples were first deproteinized with 5% 5-sulfosalicylic acid solution and centrifuged to remove the precipitated protein. Supernatant was used to measure GSH. Samples or standards (10 μL) were incubated for 5 minutes with 150 μL of working mixture (assay buffer + 5,5′-dithiobis(2-nitrobenzoic acid) + GSH reductase) in 96-well plates. Diluted NADPH solution (50 μL) was added to each well and mixed properly. The absorbance of the samples was measured at 412 nm with the kinetics for 5 minutes by using the microplate reader. The results are expressed as μM GSH/mg protein.

### Statistics

The data were analyzed for normality by assessing the sample distribution or skewness (−1.5 to +1.5 considered normally distributed) as well as homogeneity of variance using Levene’s test. After the results had passed the tests for normality, they were analyzed using one-way analysis of variance (ANOVA) followed by the post hoc Fisher’s least significant difference (LSD) test. For statistical comparisons, the software package SPSS 25.0 (IBM Middle East, Dubai, UAE) was used. The results are expressed as the means and standard errors (SEM). The *P* values less than 0.05 were considered statistically significant.

## Results

### Effects of DL77 on sociability deficits in VPA-exposed mice

The effect of subchronic systemic injection of DL77 at three different doses (5, 10, and 15 mg/kg, i.p.) and DOZ (1 mg/kg, i.p.) on ASD-like sociability deficits in the TCT task in VPA-exposed mice are shown in Fig. 2A. The results of statistical analyses revealed that subchronic treatment with DL77 (10 mg/kg, i.p.) and DOZ (1 mg/kg) prior to TCT significantly enhanced sociability by increasing time spent in the chamber of the novel mouse compared to the time spent in the chamber of the novel object, with [*F*_(7,40)_ = 4.467; *P* < 0.001] (Fig. 2A). As observed in the post hoc analysis by Fisher’s LSD test, VPA-exposed mice spent similar times in the chamber with NO and NM, with [*F*_(1,10)_ = 0.004; *P* < 0.953] compared to saline-exposed animals, which spent significantly more time with NM over NO, with [*F*_(1,10)_ = 12.20; *P* < 0.05] (Fig. 2A). DL77 (10 mg/kg) significantly improved time spent in the chamber of NM over NO in VPA-exposed mice, with [*F*_(1,12)_ = 13.375; *P* < 0.05], and the observed improvement of time spent in the chamber with NM provided by DL77 (10 mg/kg) was comparable to that shown with DOZ (1 mg/kg), with [*F*_(1,11)_ = 0.060; p = 0.810] (Fig. 2A). However, subchronic treatment of VPA-exposed mice with DL77 (5 or 15 mg/kg) failed to improve the time spent in the chamber of NM versus NO, with [*F*_(1,10)_ = 0.00; p = 0.989] and [*F*_(1,12)_ = 0.984; p = 0.341], respectively (Fig. 2A). Furthermore, neither saline + saline (group I) vs. saline + DL77 (10 mg) (group VII) nor saline + saline (group I) vs. saline + DOZ (1 mg) (group VIII) showed significant differences (p = 0.120 and p = 0.110, respectively) (Fig. 2A). In addition, a statistical analysis of observed results indicated that subchronic treatment with DL77 (10 or 15 mg/kg, i.p.) exhibited a significant sociability-enhancing effect measured as time spent exploring novel mouse [*F*_(7,43)_ = 4.98; *P* < 0.001] (Fig. 2B). As seen in Fig. 2B and in the post hoc analysis by Fisher’s LSD test, VPA-exposed mice spent similar exploratory times with NO and NM, with [*F*_(1,10)_ = 1.035; p = 0.333] compared to saline-exposed animals, which spent significantly more exploratory time with NM versus NO, with [*F*_(1,10)_ = 8.695; *P* < 0.05] (Fig. 2B). DL77 (10 or 15 mg/kg) showed significant improvements on sociability expressed as exploratory time spent with NM versus NO (both *P* values < 0.05) (Fig. 2B). However, DL77 (5 mg/kg, i.p.) and DOZ (1 mg/kg, i.p.) failed to significantly improve exploratory time spent with NM versus NO (both *P* values > 0.05) (Fig. 2B). Interestingly, neither saline + saline (group I) vs. saline + DL77 (10 mg) (group VI) nor saline + saline (group I) vs. saline + DOZ (1 mg) (group VIII) showed significant differences (p = 0.816 and p = 0.558, respectively) (Fig. 2B). As observed in the post hoc analysis by Fisher’s LSD test, the DL77-provided improvement of exploratory time spent with NM was reversed following RAMH or ZOL, with [*F*_(1,11)_ = 17.997; *P* < 0.05] or [*F*_(1,10)_ = 5.344; *P* < 0.05], respectively (Fig. 2C). However, PYR failed to reverse this sociability improvement: it had no significant effect compared to VPA-exposed mice treated with DL77 (10 mg/kg, i.p.) [*F*_(1,12)_ = 0.321; p = 0.581] (Fig. 2C). Moreover, no significant difference in the sociability index (SI) was observed for saline-exposed mice (group I) or VPA-exposed mice pretreated with 10 mg/kg of DL77 (group IV), indicating that VPA-exposed mice exhibited enhanced sociability performance when pretreated with DL77 (10 mg/kg) (Fig. 2D). Additionally, the observed results for SI values showed that the DL77 (10 mg)-provided enhancement in sociability performance was completely abrogated when co-administered with the CNS-penetrant H3R agonist RAMH (10 mg/gg, i.p.) or the CNS-penetrant H2R antagonist ZOL (10 mg/kg, i.p.) (*P* < 0.05 for the comparison of group IV with group IX or XI) (Fig. 2D). However, the CNS-penetrant H1R antagonist PYR (10 mg/kg, i.p.) failed to reverse the observed effects of DL77 (10 mg/kg) (Fig. 2D).

### Effects of DL77 on social novelty preference in VPA-exposed mice

Figure 3 shows the effects of subchronic administration of three different doses of DL77 (5, 10, and 15 mg/kg, i.p.) and DOZ (1 mg/kg, i.p.) on the time spent in the chamber with the familiar mouse (FM) and the novel mouse (NM). The results of post hoc analysis by Fisher’s LSD test indicated that saline-exposed mice spent significantly more time in the chamber of NM versus NO, with [*F*_(1,9)_ = 11.324; *P* < 0.05] (Fig. 3A). However, VPA-exposed mice spent similar times in the chamber of NM versus NO, with [*F*_(1,9)_ = 0.496; p = 0.499]. DL77 (5, 10 or 15 mg/kg, i.p.) or DOZ (1 mg/kg, i.p.) failed to provide significant differences in time spent in the chamber of NM versus FM, with [*F*_(1,10)_ = 0.150; p = 0.707], [*F*_(1,12)_ = 0.037; p = 0.851], [*F*_(1,10)_ = 0.629; p = 0.446], and [*F*_(1,10)_ = 0.061; p = 0.809], respectively (Fig. 3A). Noticeably, neither DL77 nor DOZ caused differences in time spent in the chamber with the novel mouse compared to the familiar mouse in the saline-exposed group, with [*F*_(1,12)_ = 5.784; *P* < 0.05] for saline + saline (group I) vs. saline + DL77 (10 mg) (group VII) and [*F*_(1,12)_ = 9.135; *P* < 0.05] for saline + saline (group I) + vs. saline + DOZ (1 mg) (group VIII) (Fig. 3A). In contrast, subchronic systemic pretreatment with DL77 at each dose (5, 10, or 15 mg/kg, i.p.) prior to TCT exhibited a significant enhancing effect on social novelty measured as time spent exploring NM versus FM, with [*F*_(7,40)_ = 4.67; *P* < 0.001] (Fig. 3B). As seen in Fig. 3B and in the post hoc analysis by Fisher’s LSD test, VPA-exposed mice spent similar times exploring NM and FM, with [*F*_(1,10)_ = 0.807; p = 0.390]. VPA-exposed mice pretreated with DL77 (10 or 15 mg/kg, i.p.) showed significant improvement in exploratory time spent with NM versus FM, with [*F*_(1,10)_ = 6.964; *P* < 0.05] or [*F*_(1,10)_ = 6.472; *P* < 0.05], respectively (Fig. 3B). However, DL77 (5 mg/kg, i.p.) and DOZ (1 mg/kg, i.p.) failed to improve the social novelty of VPA-exposed mice, with [*F*_(1,10)_ = 0.362; p = 0.561] and [*F*_(1,10)_ = 2.01; p = 0.187], respectively (Fig. 3B). Furthermore, no significant difference between saline + saline (group I) and saline + DL77 (10 mg) (group VII) in exploratory time for NM versus FM was found (p = 0.320) (Fig. 3B). As depicted in Fig. 3C and observed in the post hoc analysis by Fisher’s LSD test, the DL77-provided enhancement of social novelty assessed by exploratory time spent with NM versus FM was reversed by RAMH or ZOL, with [*F*_(1,10)_ = 0.331; p = 0.578] or [*F*_(1,10)_ = 0.001; p = 0.972], respectively. However, PYR failed to reverse this social novelty improvement provided with DL77 (10 mg/kg, i.p.) in treated VPA mice, since the exploratory time spent with NM versus FM remained significant following subchronic co-administration of PYR (10 mg/kg, i.p.) and DL77 (10 mg/kg, i.p.), with [*F*_(1,10)_ = 8.959; *P* < 0.05] (Fig. 3C). Moreover, no significant difference in the social novelty index (SNI) was observed between saline-exposed mice (group I) and VPA-exposed mice pretreated with 10 mg/kg of DL77 (group IV), indicating that VPA-exposed mice exhibited enhanced social novelty performance when pretreated with DL77 (10 mg/kg) (Fig. 3D). Additionally, the results for SNI values showed that the DL77 (10 mg)-provided enhancement in social novelty performance was completely counteracted when the centrally active H3R agonist RAMH (10 mg/kg, i.p.) or the CNS-penetrant H2R antagonist ZOL (10 mg/kg, i.p.) was co-administered (*P* < 0.05 for the comparison of group IV with group IX or XI) (Fig. 3D). However, the CNS-penetrant H1R antagonist PYR (10 mg/kg, i.p.) failed to reverse the enhancement of DL77-provided social novelty performance (Fig. 3D).

### Effects DL77 on stereotyped repetitive behavior of VPA-exposed mice in MBT

**Figure 4:**
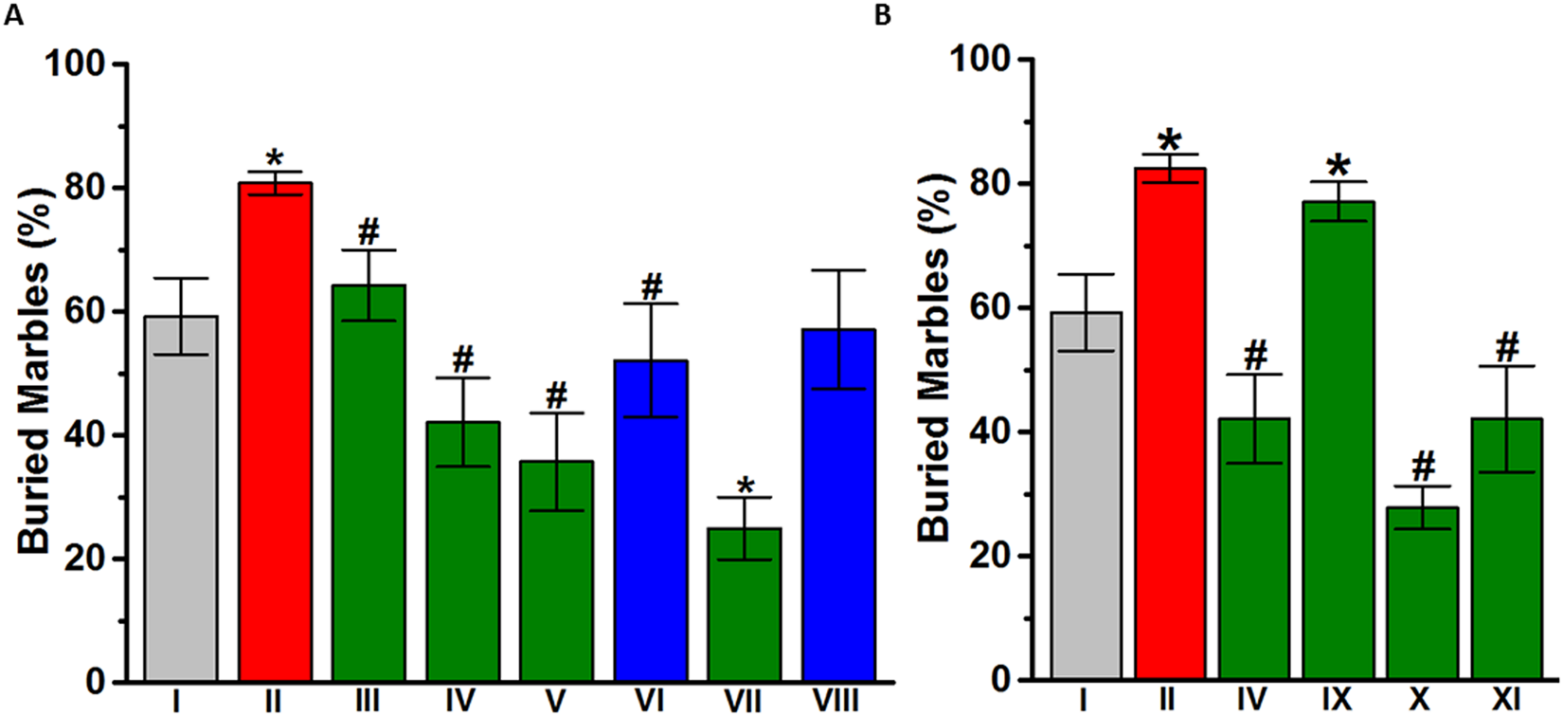
Effects of DL77 and DOZ on elevated stereotyped repetitive behavior in VPA mice exposed to MBT. (**A**) Repetitive marble-burying behavior was measured after a 30-minute testing session. VPA-exposed mice (II) demonstrated elevated stereotyped, repetitive behaviors that were significantly increased compared to saline-exposed mice (I). DL77 (at a dose of 5, 10, or 15 mg/kg in group III, IV, or V, respectively) or DOZ (1 mg/kg, i.p. in groups VI and VIII) was administered subchronically for 21 days. (**B**) Effects of sub-chronic (21 days) systemic co-administration of RAMH (10 mg/kg, i.p. for IX), PYR (10 mg/kg, i.p. for X), or ZOL (10 mg/kg, i.p., for XI) on the DL77 (10 mg)-provided attenuation of stereotyped repetitive behavior of VPA-exposed mice in MBT. Saline-exposed mice were injected with saline in group I, DL77 (10 mg/kg, i.p.) in group VII, or DOZ (1 mg/kg, i.p.) in group VIII (**A**). Figures show mean ± SEM (n = 7). *P < 0.05 vs. saline-exposed mice. ^#^*P* < 0.05 *vs*. VPA-exposed mice.

The effect of subchronic systemic injection of DL77 (5, 10, or 15 mg/kg, i.p.) or DOZ (1 mg/kg, i.p.) on the increase in repetitive behavior of VPA-exposed mice in MBT is shown in Fig. 4A,B. The results of statistical analyses showed that subchronic treatment with either 10 or 15 mg/kg DL77 or 1 mg/kg DOZ prior to MBT significantly decreased the elevated percentage of marbles buried by VPA-exposed mice [*F*_(10,64)_ = 5.128; *P* < 0.001] (Fig. 4A). As observed in the post hoc analysis by Fisher’s LSD, VPA-exposed mice buried significantly more marbles compared to the saline-exposed animals, with [*F*_(1,11)_ = 9.302; *P* < 0.05]. However, VPA-exposed mice pretreated with DL77 at 10 mg/kg or 15 mg/kg exhibited significantly decreased percentages of buried marbles compared to saline-treated VPA-exposed mice, with [*F*_(1,11)_ = 23.887; *P* < 0.001] and [*F*_(1,11)_ = 23.870; *P* < 0.001], respectively (Fig. 4A). In contrast, DL77 (5 mg/kg, i.p.) failed to significantly decrease the percentage of buried marbles in VPA-exposed mice, with [*F*_(1,11)_ = 3.674; p = 0.082] (Fig. 4A). Notably, no significant difference in the DL77-provided effect on percentage of buried marbles was observed between DL77 10 mg/kg and 15 mg/kg, with p = 0.588. Importantly, subchronic systemic administration of DOZ (1 mg/kg, i.p.) significantly decreased the percentage of marbles buried by VPA-exposed mice, with [*F*_(1,11)_ = 7.563; *P* < 0.05]. Furthermore, no significant difference in the percentage of buried marbles was observed in the comparison of saline + saline (group I) vs. saline + DOZ (1 mg) (group VIII) (p = 0.880) (Fig. 4A). However, there was a significant decrease in the percentage of buried marbles in saline-exposed mice subchronically treated with DL77 (10 mg/kg, i.p.) (group VII) vs. saline + saline (group I), with [*F*_(1,12)_ = 16.178; *P* < 0.05] (Fig. 4A). In another experiment, the DL77 (10 mg)-provided decrease in the percentage of buried marbles was reversed by RAMH, with [*F*_(1,11)_ = 1.499; p = 0.246] for the comparison of VPA-exposed mice vs. VPA-exposed mice + DL77 (10 mg) + RAMH(10 mg). However, PYR and ZOL failed to reverse this DL77 (10 mg)-provided decrease in the percentage of buried marbles, as they had no significant effect compared to VPA-exposed mice treated with DL77 (10 mg/kg, i.p.), with [*F*_(1,11)_ = 135.98; *P* < 0.001] and [*F*_(1,11)_ = 15.311; *P* < 0.05], respectively (Fig. 4B).

### Effects DL77 on stereotyped repetitive behavior of VPA-exposed mice in NST

**Figure 5:**
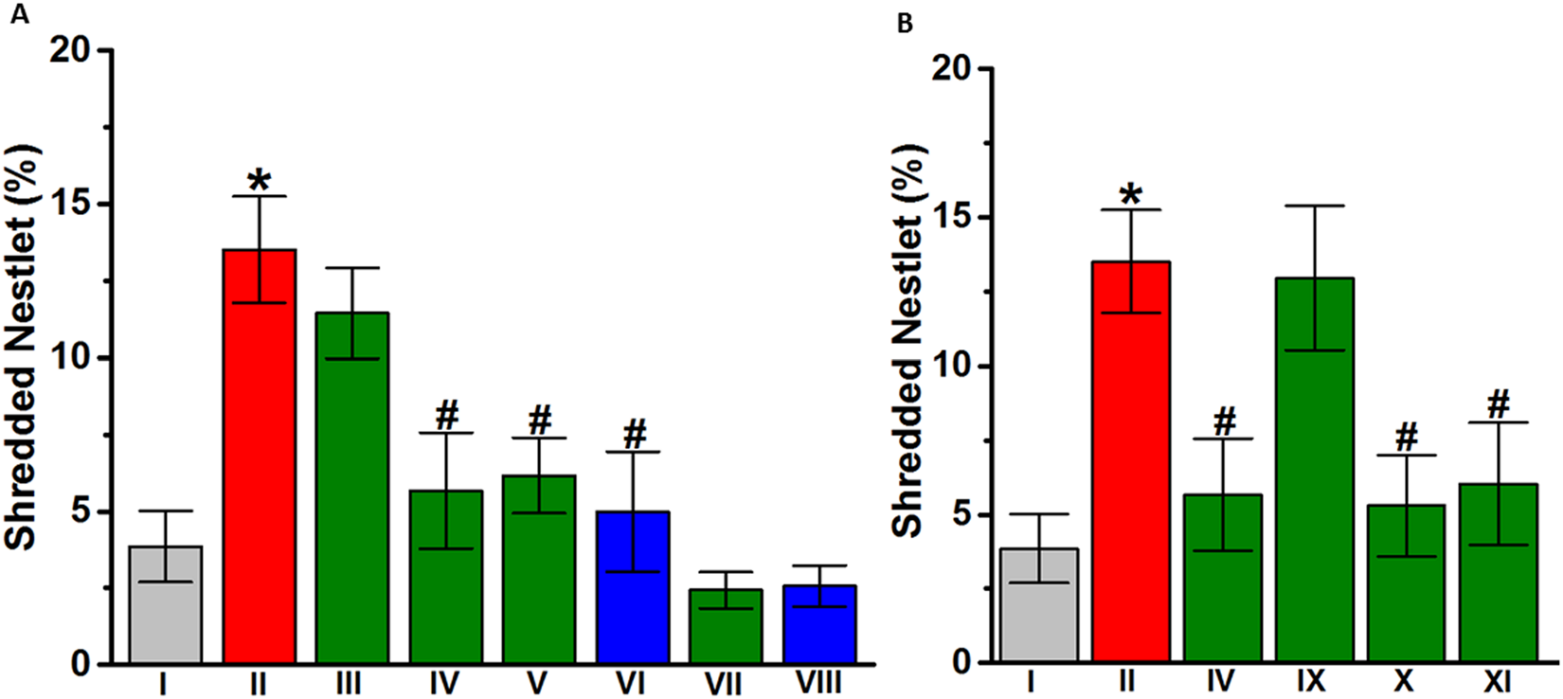
Effects of DL77 and DOZ on elevated stereotyped repetitive behavior in VPA mice exposed to NST. (**A**) Repetitive nestlet-shredding behavior was measured after a 30-minute testing session. VPA-exposed mice (II) demonstrated elevated stereotyped repetitive behaviors that were significantly increased compared to saline-exposed mice (I). DL77 (at a dose of 5, 10, or 15 mg/kg in group III, IV, or V, respectively) or DOZ (1 mg/kg, i.p. in groups VI and VIII) was administered sub-chronically for 21 days. (**B**) Effects of subchronic (21 days) systemic co-administration of RAMH (10 mg/kg, i.p. for IX), PYR (10 mg/kg, i.p. for X), or ZOL (10 mg/kg, i.p., for XI) on DL77 (10 mg)-provided attenuation of stereotyped repetitive behavior of VPA-exposed mice in NST. Saline-exposed mice were injected with saline in group I, DL77 (10 mg/kg, i.p.) in group VII, or DOZ (1 mg/kg, i.p.) in group VIII (**A**). Figures show mean ± SEM (n = 7). *P < 0.05 vs. saline-exposed mice. ^#^*P* < 0.05 *vs*. VPA-exposed mice.

In the current study, the effect of subchronic systemic injection of DL77 (5, 10, or 15 mg/kg, i.p.) or DOZ (1 mg/kg, i.p.) on the increase in the percentage of shredded nestlet was assessed in NST (Fig. 5A,B). As observed in the post hoc analysis by Fisher’s LSD test, VPA-exposed mice shredded significantly more nestlet compared to the saline-exposed mice, with [*F*_(1,10)_ = 17.851; *P* < 0.05]. VPA-exposed mice treated with DL77 (10 mg/kg or 15 mg/kg) exhibited a significantly lower percentage of shredded nestlet compared to VPA-exposed mice treated with saline, with [*F*_(1,11)_ = 7.717; *P* < 0.05] and [*F*_(1,11)_ = 10.549; *P* < 0.05], respectively (Fig. 5A). However, DL77 (5 mg/kg) failed to modulate the percentage of shredded nestlet in VPA-exposed mice, with [*F*_(1,11)_ = 0.714; p = 0.416]. Notably, no significant difference in the DL77-provided effect on percentage of shredded nestlet was observed between the two doses of 10 and 15 mg/kg, with p = 0.842. Moreover, subchronic systemic administration of DOZ (1 mg/kg, i.p.) significantly lowered the percentage of shredded nestlet in VPA-exposed mice, with [*F*_(1,11)_ = 8.869; *P* < 0.05]. Furthermore, neither saline + saline (group I) vs. saline + DL77 (10 mg) (group VII) nor saline + saline (group I) vs. saline + DOZ (1 mg) (group VIII) showed significant differences in shredded nestlet (p = 0.467 and p = 0.401, respectively) (Fig. 5A). As depicted in Fig. 5B and observed in the post hoc analysis by Fisher’s LSD test, the DL77 (10 mg)-provided decrease in the percentage of shredded nestlet was reversed following RAMH, with [*F*_(1,11)_ = 0.028; p = 0.871] compared with saline-treated VPA-exposed mice (Fig. 5B). However, PYR and ZOL failed to reverse this DL77-provided decrease in the percentage of shredded nestlet, as they had no significant effect compared to VPA-exposed mice treated with DL77 (10 mg/kg, i.p.), with [*F*_(1,11)_ = 9.532; *P* < 0.05] and [*F*_(1,11)_ = 6.291; *P* < 0.05], respectively (Fig. 5B).

### Effects of DL77 on anxiety and locomotor activity of VPA-exposed mice in EPM

**Figure 6:**
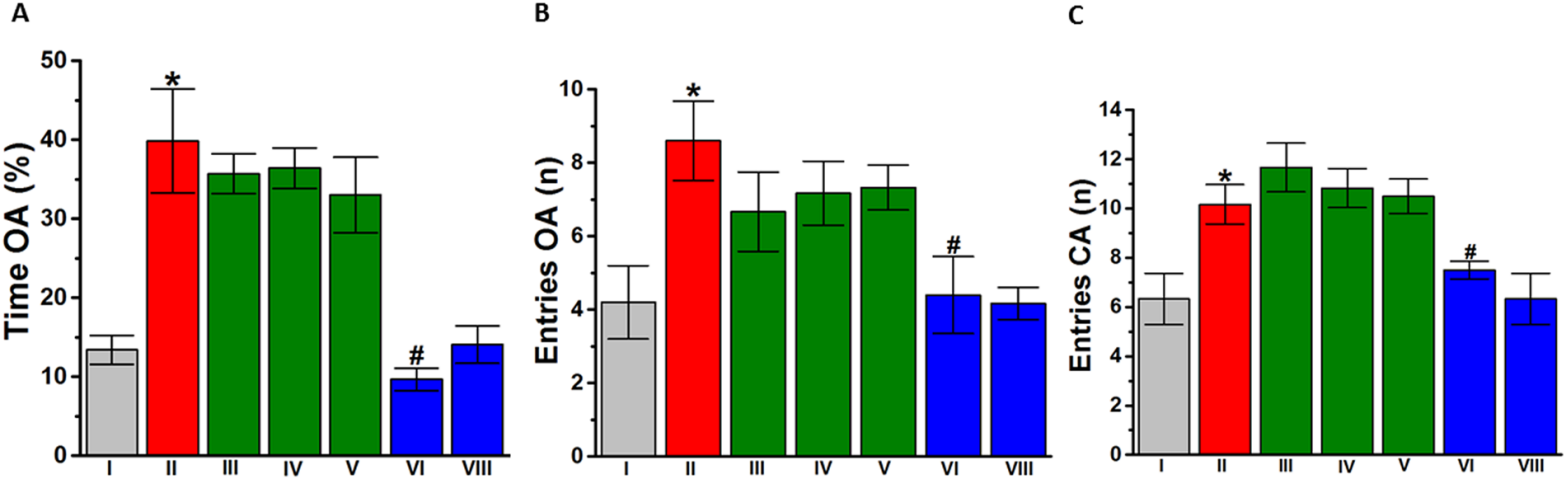
Effects of DL77 and DOZ pretreatment on exploratory behavior in the EPM test. VPA-exposed mice (II) demonstrated elevated impulsive attitude and deficits in cognition behaviors that were significantly increased compared to saline-exposed mice (I). DL77 (5, 10, or 15 mg/kg, i.p. in group III, IV, or V, respectively) or DOZ (1 mg/kg, i.p., in group VI) was administered subchronically for 21 days. DOZ (1 mg/kg, i.p. in groups VI and VIII) attenuated the increased percentage of time spent on the open arms of the EPM (**A**) the increased number of entries into the open arms (**B**) and the increased number of entries into the closed arms (**C**) in VPA mice. However, pretreatment with the H3R antagonist DL77 (5, 10, or 15 mg/kg, i.p.) did not affect any of the three parameters (**A**–**C**). Saline-exposed mice were injected with saline in group I and DOZ (1 mg/kg, i.p.) in group VIII (**A**–**C**). Data are expressed as the mean ± SEM (n = 5–7). *P < 0.05 vs. saline-exposed mice. ^#^*P* < 0.05 *vs*. VPA-exposed mice.

Figure 6 shows the observed effects of subchronic systemic injection of saline or the H3R antagonist DL77 (5, 10, or 15 mg/kg, i.p.) on the anxiety parameters of VPA-exposed mice tested in the EPM, namely, the percentage of time spent in open arms (Fig. 6A), the number of entries into open arms (Fig. 6B), and locomotor activity expressed as the number of entries into closed arms (Fig. 6C). Subsequent post hoc analyses showed that DL77 administered at 5, 10, or 15 mg/kg i.p. did not alter the percentage of time spent exploring the open arms of the maze during a 5 min session compared to saline-treated VPA-exposed mice, with [*F*_(1,9)_ = 0.301; p = 0.596], [*F*_(1,9)_ = 0.178; p = 0.683], or [*F*_(1,9)_ = 0.563; p = 0.472], respectively (Fig. 6A). However, VPA-exposed mice pretreated with DOZ (1 mg) spent a significantly lesser percentage of time exploring the open arms compared to saline-treated VPA-exposed mice, with [*F*_(1,7)_ = 11.835; *P* < 0.05] (Fig. 6A). Further analyses of data describing the number of entries and percentage of time in the open arms of the maze yielded for DL77 (5, 10, and 15 mg/kg i.p.) and DOZ (1 mg/kg, i.p.) practically the same results, with [*F*_(1,9)_ = 1.746; p = 0.219], [*F*_(1,9)_ = 1.199; p = 0.302], [*F*_(1,9)_ = 1.211; p = 0.299], and [*F*_(1,7)_ = 7.217; *P* < 0.05], respectively (Fig. 6B). Interestingly, the number of closed arm entries following subchronic systemic injection of DL77 (5, 10, or 15 mg/kg) was not significantly different, with [*F*_(1,10)_ = 0.990; p = 0.343], [*F*_(1,10)_ = 0.292; p = 0.601], and [*F*_(1,10)_ = 0.059; p = 0.813], receptively. However, DOZ (1 mg)-treated VPA-exposed mice (group VI) showed fewer closed arm entries compared to saline-treated VPA-exposed mice (group II), with [*F*_(1,10)_ = 6.598; *P* < 0.05] (Fig. 6C).

### Effect of DL77 on oxidative stress levels in brain tissue of VPA-exposed mice

**Figure 7:**
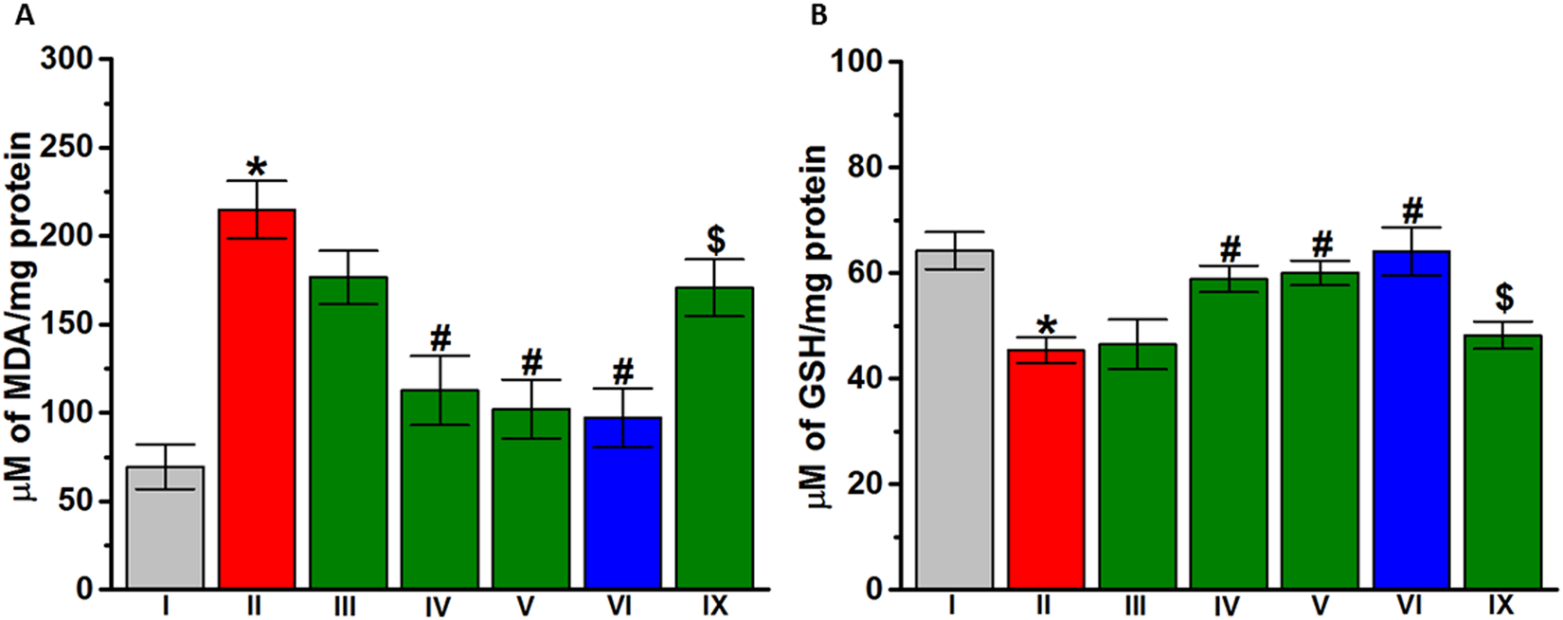
Effects of DL77 and DOZ pretreatment on levels of malondialdehyde (MDA) and glutathione (GSH) in the cerebellum. VPA-exposed mice (II) showed a significant increase in MDA (**A**) and significant decrease in GSH (**B**) compared to saline-exposed mice (I). DL77 (5, 10, or 15 mg/kg, i.p. in group III, IV, or V, respectively) or DOZ (1 mg/kg, i.p. in group VI) was administered subchronically for 21 days. DL77 (10 or 15 mg/kg, i.p. in group IV or V, respectively) or DOZ (1 mg/kg, i.p. in group VI) significantly decreased MDA and significantly increased GSH. RAMH (10 mg/kg, i.p., co-administered in group IX) abrogated the modulating DL77 (10 mg)-provided effects on oxidative stress levels (**A**,**B**). Saline-exposed mice were injected with saline in group I (**A**,**B**). Data are expressed as the mean ± SEM (n = 5–7). *P < 0.05 vs. saline-exposed mice. ^#^*P* < 0.05 vs. VPA-exposed mice. ^$^*P* < 0.05 vs. DL77 (10 mg)-treated VPA-exposed mice.

The ability of DL77 to reduce oxidative stress in VPA-exposed mice was evaluated. Two parameters were measured, the MDA and GHS levels in VPA-exposed mouse cerebellum, which responded in an exacerbated way to the inflammatory stimulus LPS. The results in Fig. 7A,B show that MDA was significantly elevated (*P* < 0.001) and GHS was significantly decreased (*P* < 0.05) in the brain tissues of VPA-exposed mice compared to saline-exposed mice (Fig. 7A,B). However, brain tissues of VPA-exposed mice subchronically pretreated with DL77 (10 or 15 mg/kg, i.p.) or DOZ (1 mg/kg, i.p.) showed a significant reduction of MDA (from 214.88 ± 12.58 μM to 112.77 ± 19.56, 102.24 ± 16.60, and 97.32 ± 16.55 μM for DL77 (10 and 15 mg/kg) and DOZ (1 mg/kg), respectively) (*P* < 0.05) (Fig. 7A). In addition, subchronic administration of DL77 (10 or 15 mg/kg) or DOZ (1 mg/kg) significantly increased GHS compared to the saline-treated VPA-exposed animals (Fig. 7B). Meanwhile, the VPA-exposed mice subchronically pretreated with 5 mg/kg did not show any significant difference from the saline-treated VPA-exposed group (Fig. 7A,B). Moreover, subchronic systemic co-administration of RAMH (10 mg/kg, i.p.) partially abrogated (*P* < 0.005) the protective effects of DL77 (10 mg/kg, i.p.) against the VPA-induced decreased level of MDA (Fig. 7A), and it completely reversed the DL77 (10 mg)-provided increase in GHS in VPA-exposed mice (Fig. 7B).

### Effects of DL77 pretreatment on levels of proinflammatory cytokines IL-1β, IL-6 and TNF-α in brain tissue of VPA-exposed mice

**Figure 8:**
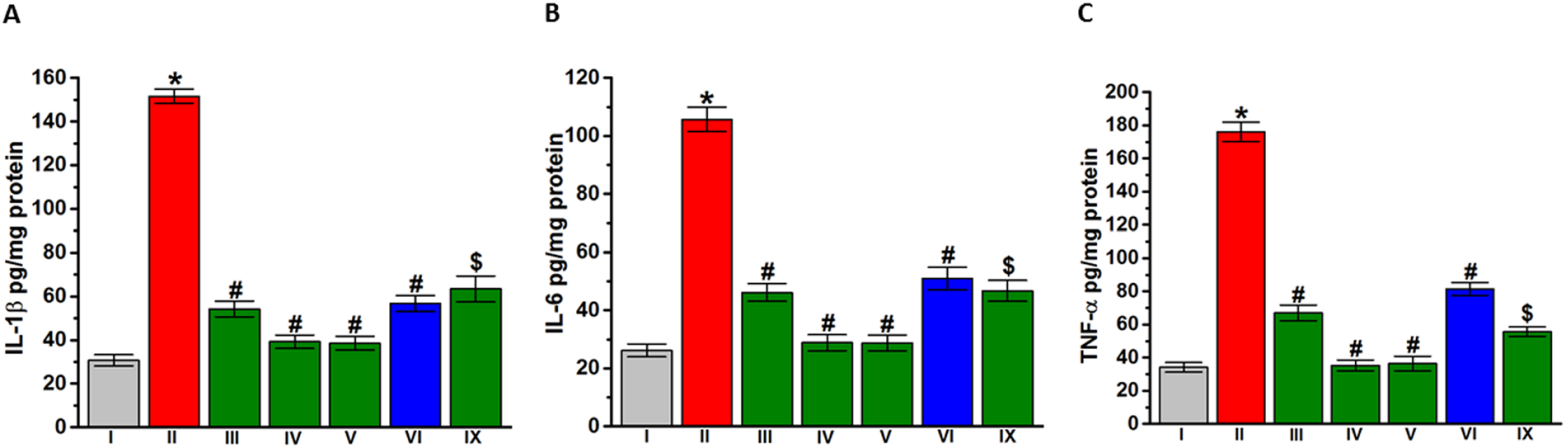
Effects of DL77 and DOZ pretreatment on levels of proinflammatory cytokines IL-1β, IL-6 and TNF-α in the cerebellum. VPA-exposed mice (II) showed significantly increased IL-1β (**A**) IL-6 (**B**) and TNF-α (**C**) compared to saline-exposed mice (I) exacerbated by LPS challenge. DL77 (5, 10, or 15 mg/kg, i.p. in group III, IV, or V, respectively) or DOZ (1 mg/kg, i.p. in group VI) was administered subchronically for 21 days. DL77 (5, 10, or 15 mg/kg, i.p. in group III, IV, or V, respectively) or DOZ (1 mg/kg, i.p. in group VI) significantly decreased IL-1β (**A**), IL-6 (**B**) and TNF-α (**C**). RAMH (10 mg/kg, i.p., co-administered in group IX) reversed the modulating DL77 (10 mg)-provided effects on proinflammatory cytokines (**A**–**C**). Saline-exposed mice were injected with saline in group I (**A**–**C**). Data are expressed as the mean ± SEM (n = 5–7). *P < 0.05 vs. saline-exposed mice. ^#^*P* < 0.05 vs. VPA-exposed mice. ^$^*P* < 0.05 vs. DL77 (10 mg)-treated VPA-exposed mice.

The effects of DL77 on the levels of proinflammatory cytokines IL-1β, IL-6 and TNF-α in brain tissue of VPA-exposed mice that were exacerbated with LPS challenge were also assessed (Fig. 8A–C). The induction of ASD-like behaviors by prenatally administered VPA significantly increased IL-1β (Fig. 8A), IL-6 (Fig. 8B), and TNF-α (Fig. 8C) compared to the saline-exposed mice (all *P* < 0.001), while subchronic systemic administration of DL77 (5, 10 or 15 mg/kg, i.p.) or DOZ (1 mg/kg) significantly (*P* < 0.001) attenuated the rise of these proinflammatory cytokines in VPA-exposed mice (Fig. 8A–C). Notably, subchronic systemic co-administration of RAMH (10 mg/kg, i.p.) partially abrogated (*P* < 0.005) the protective effects of DL77 (10 mg/kg, i.p.) against VPA-induced elevation of proinflammatory cytokines (Fig. 8A–C).

## Discussion

Antagonists targeting H3Rs are considered promising alternative treatments for different brain disorders, such as SCH, AD and narcolepsy^9,51^. The current study investigated, for the first time, the effects of the H3R antagonist DL77 on a mouse model of ASD-like behaviors induced by prenatal exposure to VPA. DL77 was chosen for the current study because it belongs to the class of non-imidazole H3R antagonists with improved selectivity and safety profile compared to the imidazole-based H3R antagonists, e.g., ciproxifan, which have several limitations as clinical candidates due to the presence of an imidazole heterocycle, which is responsible for numerous possible pharmacokinetic drawbacks, such as CYP450 inhibition, off-target activity, or lack of subtype selectivity, especially over H4R^51^.

Subchronic systemic administration of DL77 demonstrated ameliorating effects on social interaction deficits and stereotypies in VPA-exposed mice. In the TCT paradigm, sociability or social novelty is the tendency to spend time exploring an unfamiliar animal, compared to time spent exploring an object or a familiar animal, respectively. Systemic subchronic pretreatment with DL77 normalized the impairment in sociability demonstrated by VPA-exposed mice, since these animals, when pretreated with DL77, presented longer times spent in the chamber of the novel mouse (improved sociability, Fig. 2B,D) and increased time spent exploring the novel mouse (improved social novelty, Fig. 3B,D), to levels similar to the saline-exposed mice. Numerous previous studies have focused on the procognitive effects of several H3R antagonists on social memory^24,52–57^, a behavioral feature that is also altered in ASD^58^, but this study is the first one to assess the effects on of a non-imidazole based H3R antagonist on sociability deficits as ASD-like behaviors in VPA-exposed mice. Importantly, the sociability- and social novelty-enhancing effect observed for DL77 was dose-dependent, since DL77 (5 mg/kg) failed to improve sociability (Fig. 2B,D) or social novelty preference (Fig. 3B,D), while a dose of 10 mg/kg or 15 mg/kg provided significant enhancement in sociability as well as social novelty behavior. An optimum effect was observed when the H3R antagonist DL77 was applied at a dose of 10 mg/kg, and a dose of 15 mg/kg DL77 did not significantly improve upon the DL77 (10 mg)-provided sociability or social novelty enhancement (Figs 2B,D and 3B,D). Interestingly, the observations for the dose-dependent effects of DL77 are in line with those previously observed for numerous H3R antagonists in preclinical experiments in different rodents (Benetti and Izquierdo, 2013; Benetti *et al*., 2013; Sadek *et al*., 2016c). In addition, the observed results in regard to dose dependency strongly support our previous procognitive effects observed for the H3R antagonist DL77 (2.5, 5, or 10 mg/kg, i.p.) on different memory stages, namely, acquisition, consolidation, and retrieval in rats (Sadek *et al*., 2016c). Notably, the observations of sociability- and social novelty-enhancing effects for DL77 are in agreement with earlier experimental results observed with the imidazole-based H3R antagonist ciproxifan in Swiss mice^1^. In that study, ciproxifan (3 mg/kg, i.p.) attenuated sociability deficits in the VPA-induced ASD-like behaviors (Diego Baronio *et al*.^1^).

Interestingly, the DL77 (10 mg)-provided enhancing effects on sociability and social novelty were completely reversed when mice were co-administered the CNS-penetrant H3R agonist RAMH or with the CNS-penetrant H2R antagonist ZOL, but not with the centrally acting H1R antagonist PYR (Figs 2C,D and 3C,D). The latter observations indicate that brain histaminergic neurotransmission appears to be involved in the capacity of the H3R antagonist DL77 to facilitate the release brain histamine in specific brain areas^7,9,10,59^. The current results further indicate that histaminergic pathways, through activation of H2Rs, fundamentally contribute to neuronal pathways important for alteration of sociability and social novelty processes in the TCT paradigm in VPA-exposed mice. Notably, the reference drug DOZ failed to improve the exploratory time spent with the novel mouse compared to the time spent with the novel object (Fig. 2B,D). Additionally, DOZ did not improve the social novelty preference of VPA-exposed mice following subchronic systemic treatment (Fig. 3B,D). However, subchronic systemic pretreatment with DOZ significantly enhanced the time spent in the chamber of the novel mouse, indicating that sociability measures of VPA-exposed mice were improved after subchronic systemic treatment with DOZ (1 mg/kg) (Fig. 2A). These observations could be explained by the results of a previous study in which the integrity of the central histaminergic system was found to be a crucial requirement for the biochemical and behavioral effects elicited by two procognitive compounds, namely, the H3R antagonist ABT-239 and the acetylcholine esterase inhibitor DOZ^34^. Consequently, further investigations are necessary to assess the functionality of the central histaminergic system in VPA-exposed mice to further explain the behavioral results observed for DOZ in sociability as well as social novelty tests.

Stereotypy and rigidity of behavior are considered core features of ASD^60,61^. Moreover, the involvement of the brain histaminergic neurotransmission in the pathophysiology of Tourette syndrome, a condition commonly comorbid among ASD patients and featured by stereotypies, has been proposed and has been associated with a premature termination codon (W317X) responsible for the L-histidine decarboxylase (HDC) gene, the rate-limiting enzyme in biosynthesis of brain histamine. Consequently, such a premature termination of codon (W317X) impairs brain histaminergic neurotransmission^16,62–65^. In the current study, VPA-exposed mice subchronically pretreated with DL77 (10 or 15 mg/kg, i.p.) or with the reference drug DOZ (1 mg/kg, i.p.) displayed comparable reductions in stereotyped repetitive behavior in MBT (Fig. 4A). Moreover, the DL77 (10 mg/kg)-provided effects in MBT were entirely abrogated when mice were administered the CNS penetrant H3R agonist RAMH, but not with the CNS-penetrant H1R antagonist PYR or the H2R antagonists ZOL (Fig. 4B). The mechanism by which the repetitive behavior is improved is not clear, but it might be explained by the capability of DL77, as a potent H3R antagonist, to mediate the release of different neurotransmitters besides histamine, such as dopamine, serotonin and acetylcholine, in several specific brain areas^7,9,12,59^. Therefore, measuring the levels of different brain neurotransmitters, including histamine, in various brain areas of the VPA-exposed mice with ASD-like behaviors as well as when treated with DL77 would further help us understand which neural circuits could be involved in this observed behavioral improvement. Interestingly, the results observed for DL77 in MBT are in agreement with a previous study in which acute systemic administration of the non-imidazole H3R antagonist ST-1283 significantly decreased the number of buried marbles and significantly shortened the digging duration in adult male C57BL/6 mice, without altering locomotor activity tested in an open field^42^.

Similarly, DL77 (10 or 15 mg/kg) attenuated the percentage of shredded nestlet in VPA-exposed mice with ASD-like behaviors in NST, and this reducing effect of DL77 on stereotyped repetitive behavior in NST was reversed with co-administration of the H3R agonist RAMH, but not the H1R antagonist PYR or the H2R antagonist ZOL (Fig. 5A,B). The results observed for DL77 in NST explain the current observations in MBT because DL77 facilitates the release of numerous central neurotransmitters besides histamine that are involved in repetitive as well as anxious behaviors in different rodents^7,9,12,59^.

Ligands modulating anxiety levels or locomotion may give rise to a false-positive effect in these behavioral tests^42,66^. Therefore, the numbers of entries into the closed arms and into open arms in the EPM test were used as indicators of locomotor activity and anxiety levels, respectively. The results showed that DL77 at a dose of 5, 10 or 15 mg/kg mg/kg did not alter anxiety-like levels nor locomotor activity in VPA-exposed mice as measured by the percentage of time spent or the number of entries into open arms and closed arms, respectively (Fig. 6A–C). Thus, the improvements in sociability, social novelty, and repetitive behaviors observed for DL77 in TCT, MBT and NST, respectively, appear unlikely to be associated with a modulating effect in anxiety levels or an increase in locomotor activity of the tested mice. In contrast, the reference drug DOZ (1 mg/kg, i.p.) attenuated the percentage of time spent and the number of entries into open arms, indicating its enhancing effects on cognitive functions of VPA-exposed mice to better recognize and evaluate anxiety, as reflected in the decreased number of entries into open arms following subchronic systemic pretreatment VPA-exposed mice with DOZ (Fig. 6A–C). Moreover, the failure of DL77 at all doses to improve this abnormal anxiety and hyperactivity seen in VPA-exposed mice may have been due to the imbalance of several neurotransmitters that are dysregulated in ASD patients, such as serotonin^67,68^, glutamate and GABA^68–71^.

Several hypotheses have been proposed about the dual role of brain histamine in neurological disorders, and evidence has shown its critical involvement in the modulation of microglia-mediated neuroinflammation^72^. To determine a possible function of DL77 in tissue defense, oxidative stress parameters MDA and GSH were assessed in the cerebellum (Fig. 7A,B). The existence of lipid peroxidation is considered a key pathogenic event in brain tissues, resulting from an unbalanced ratio of radical oxygen species generation and the capacity for endogenous cellular antioxidant defense^73^. The results showed that VPA-exposed mice with ASD-like behaviors had a significant increase in MDA, with a concomitant decline in GSH in the cerebellum tissues. GSH is considered to fight against lipid peroxidation, and it could have been depleted in the oxidative stress^74^, as demonstrated in the observed results (Fig. 7A,B). The results of the current study are in accordance with recent findings that histamine may provide protective effects under an inflammatory context^72^. However, subchronic systemic pretreatment with DL77 (10 or 15 mg/kg, i.p.) inhibited lipid peroxidation, as evidenced by the reduced MDA followed by restoration of GSH (Fig. 7A,B). Interestingly, the imidazole-based H3R antagonists, namely, ciproxifan and clobenpropit, control the elevation of various oxidative stress markers, including MDA and GSH, in amphetamine- or dizocilpine-augmented oxidative stress in an experimental mice model of SCH, indicating that H3R antagonists possess antioxidant activity, which might serve the antioxidant needs of SCH and at the same time control the symptomatic features of SCH^75,76^. These results in an SCH experimental model strongly support the current observations regarding DL77 in VPA-exposed mice with ASD-like behaviors, since SCH and ASD are often comorbid and share several symptomatic features^9,76^.

The protective role of histamine has also been reported in neurological conditions featured by microglia-induced neuroinflammation and, consequently, the involvement of brain histamine in the pathophysiology of multiple sclerosis and other neurological diseases of several animal models^72,77^. The current study demonstrated a significant LPS-induced exacerbated rise in the expression of proinflammatory cytokines (IL-1β, IL-6, and TNF-α) in VPA-exposed mice (Fig. 7A–C). However, subchronic systemic pretreatment with DL77 (5, 10, or 15 mg/kg, i.p.) significantly decreased the elevated levels of these proinflammatory cytokines in VPA-exposed mice. Interestingly, the CNS-penetrant H3R agonist, when co-administered with DL77, completely reversed the protective effect of DL77 against the elevated oxidative stress and partially abrogated the DL77-provided protective effects on proinflammatory cytokines (Fig. 7A–C), indicating the involvement of central histaminergic neurotransmission in mediating the neuroprotective action of DL77 in VPA-exposed mice with ASD-like behaviors under inflammatory environments.

## Conclusion

In summary, subchronic systemic administration of the non-imidazole H3R antagonist DL77 palliated sociability deficits and stereotypies present in the animal model of ASD-like behaviors induced by prenatal VPA exposure, demonstrating the therapeutic potential of H3R antagonists in the treatment of ASD. Moreover, modulation of central histaminergic neurotransmission by the H3R antagonist DL77 in an inflammatory context may have attenuated proinflammatory cytokine release and oxidative stress levels. However, additional preclinical experiments in other behavioral test models and with several rodent species are necessary to expand these initial data and to understand the translational potential of H3R antagonists in the therapy of ASD.
